# Sedentary antlion larvae (Neuroptera: Myrmeleontidae) use vibrational cues to modify their foraging strategies

**DOI:** 10.1007/s10071-016-1000-7

**Published:** 2016-05-24

**Authors:** Karolina Kuszewska, Krzysztof Miler, Michał Filipiak, Michal Woyciechowski

**Affiliations:** Institute of Environmental Sciences, Jagiellonian University, Kraków, Poland

**Keywords:** Antlion, Associative learning, Foraging strategy, Rescue behaviour, Ambush predator

## Abstract

Learning abilities are exhibited by many animals, including insects. However, sedentary species are typically believed to have low capacities and requirements for learning. Despite this view, recent studies show that even such inconspicuous organisms as larval antlions, which employ an ambush predation strategy, are capable of learning, although their learning abilities are rather simple, i.e., limited to the association of vibrational cues with the arrival of prey. This study demonstrates, for the first time, that antlion larvae can use vibrational cues for complex modifications of their foraging strategies. Specifically, antlion larvae rapidly learn to differentiate between the vibrational cues associated with prey of different sizes, and they save resources by ignoring smaller prey in favour of larger, more energetically profitable prey. Moreover, antlion larvae can learn to associate vibrational cues with the loss of their prey, and they respond by burying their victims under the sand more often and more rapidly than do individuals with no opportunities to form such associations. These findings provide not only new insights into the cognitive abilities of animals but also support for the optimal foraging strategy concept, suggesting the importance of maximizing fitness output by balancing the costs and benefits of alternative foraging strategies.

**Electronic supplementary material:**

The online version of this article (doi:10.1007/s10071-016-1000-7) contains supplementary material, which is available to authorized users.

## Introduction

Understanding the mechanisms of animal associative learning and memory is an important aspect of neurobiology and behavioural ecology (Thompson 1986; Dickinson 2012). The ability to learn allows individuals to adjust their behaviour to changing environmental conditions, which can have a considerable impact on individual fitness components (Dukas 2000; Hollis et al. 2004). Research concerning associative learning is usually focused on vertebrates, but several studies have presented evidence of associative learning in insects (e.g., Dukas 2008; Hollis and Guillette 2011; Giurfa 2013). Such studies have shown similar behavioural patterns in insects belonging to very different groups. These patterns include active search for food, hosts, and/or mates, as well as active predator avoidance. It is believed that associative learning improves the efficiency of active search (e.g., Behmer et al. 1999; Chilaka et al. 2012). However, there is also evidence that sedentary insects, such as the larvae of pit-building antlions (Neuroptera: Myrmeleontidae), are capable of associative learning. These animals construct funnel-shaped pitfall traps under the sand (Turner 1915) and their predatory strategy consists of waiting for prey to stumble into their trap (Scharf and Ovadia 2006; Scharf et al. 2008). A recent study showed that antlion larvae more frequently respond to vibrational cues through head and mandible movements after learning to associate cues with the arrival of prey (Guillette et al. 2009). The learned individuals also moult significantly sooner than do non-learned antlions, resulting in decreased time spent by the former in the vulnerable larval stage (Hollis et al. 2011, 2015).

The foraging strategies of antlion larvae are diverse and depend extensively on environmental conditions. Individuals can flexibly alternate between foraging with trap and prey ambush with no trap according to their energy status. When their energy status is high (i.e., they are well fed), antlion larvae use the ambush strategy, whereas the pit-trap strategy is used when their energy status is low (i.e., when they are hungry; Tsao and Okuyama 2012). The rate of prey encounter can also modify the strategy employed: when prey abundance is high, antlions reduce both prey handling time and the percentage of nutrients extracted from each prey and increase their rate of prey ingestion (Lucas 1985). Thus, antlions respond to changes in their environment in ways that maximize their fitness, consistent with optimal foraging strategy theory, which predicts that foraging organisms will improve their fitness by maximizing net energy intake per unit time and will typically choose the available food type that yields the highest energetic gain per catch effort (Arnett and Gotelli 2001; Scharf and Ovadia 2006).

Associative learning can lead to significant enhancement of the foraging performance of animals. Choosing the optimal foraging strategy, however, is closely associated with the ability to learn the cues associated with the arrival of different prey types. Thus, we investigated whether pit-building antlion larvae can learn cues that differ in intensity that are associated with various events and whether they can change their foraging strategy depending on the cue perceived. We performed two experiments to examine whether antlions possess such capabilities.

## Experiment 1

In Experiment 1, we explored whether antlions can associate small vibrational cues with the arrival of small-sized prey and large vibrational cues with the arrival of larger prey and then use these associations to modify their foraging strategy. We assumed that large prey are generally preferred by antlions because larvae grow faster when fed large prey (Alcalay et al. 2014). For this purpose, we paired eighty antlions by weight, with one larva from each pair randomly assigned to the trained treatment and the other assigned to the untrained (control) treatment (see also Supplementary Methods). The experiment consisted of a training phase followed by a test phase; the training phase consisted of 3 blocks, each lasting for 3 days (2 consecutive training days followed by a 1-day rest). Trained and untrained antlions were provided with prey in the centre of their pits 4 times per day at 2-h intervals between 10 AM and 6 PM. On each training day, small prey was provided in 2 of the 4 feeding incidents in random order, with large prey provided at the other 2 feeding incidents. Both trained and untrained antlions were fed at the same time. For trained antlions, prey was delivered immediately after an associated vibrational cue (small cue for small prey; large cue for large prey), whereas for untrained antlions, the vibrational cue was presented not directly preceding prey delivery but either 5–10 min before prey delivery or 5–10 min after (randomly selected). We used the drop of 3 ml of sand as the small cue and the drop of 6 ml of sand as the large cue. This setup was prepared similarly to setups described in previous research (Guillette et al. 2009; Hollis et al. 2011, 2015). For the test phase, the 40 trained-untrained pairs of antlions were randomly divided into two groups, each consisting of 18 trained–untrained pairs (4 pairs were excluded due the absence of functional pitfall traps). In the first group, all trained and untrained antlions received the small cue, followed by the provision of small prey; after a 30-s period during which antlions captured their small prey, the large cue was delivered. In the second group, all of the trained and untrained antlions received large prey preceded by the large cue and followed by the small cue. In both groups, we noted whether the captured prey was buried under the sand or rejected, and if either occurred, then the time of burial or rejection within the 3 min after the second cue was delivered was recorded.

**Fig. 1:**
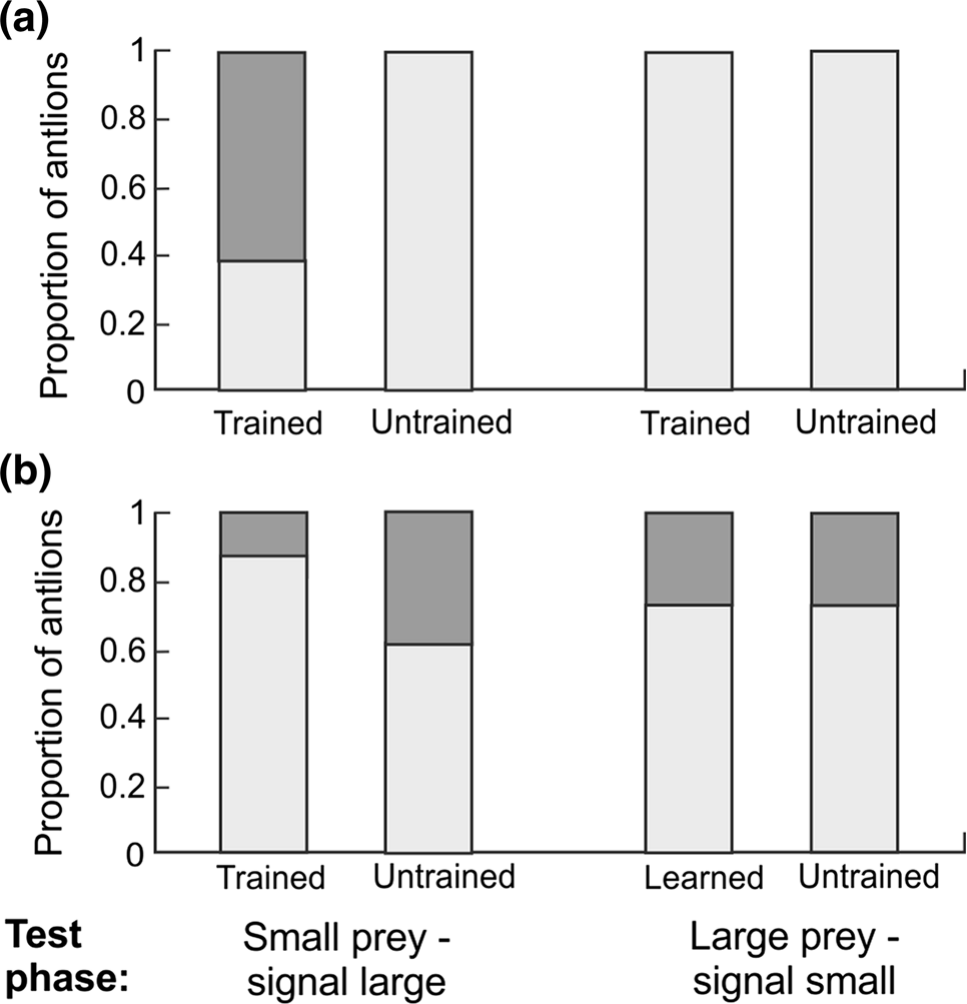
Behaviour of antlions trained to associate small/large cues with small/large prey items and of untrained antlions with no opportunity to form such associations. **a** Proportion of antlions rejecting (*dark grey bars*) and not rejecting prey from the pit-trap (*light grey bars*) during the 3 min following the second cue presentation; **b** proportion of antlions initiating burial (*dark grey bars*) and not initiating burial (*light grey bars*) of their victims during the 3 min following the second cue presentation

The results show that of the 18 trained antlions in Experiment 1, 11 (61 %) rejected small prey immediately after the cue associated with large prey was given, this behaviour was not observed in the untrained groups (Fisher’s exact test: *p* = 0.0001; Bonferroni correction *p* < 0.008; Fig. 1a). The median time of rejection in trained antlions was 10 s (quartiles 6–21 s). There was no difference between trained and untrained antlions in large prey rejection following the small cue (Fisher’s exact test: *p* = 1.0000; Bonferroni correction *p* < 0.008). None of the antlions in either the trained or untrained group rejected the large prey (Fig. 1a). In addition, no difference in the proportion of buried victims between the learned and control antlions was detected (Fisher’s exact test: *p* > 0.1774; Bonferroni correction *p* < 0.008; Fig. 1b).

## Experiment 2

In Experiment 2, we tested whether antlions can learn that a vibrational cue is associated with the loss of prey and whether antlions will act to prevent prey loss after such learning. The setup used was similar to that described for Experiment 1; however, we used 60 weight-matched antlions (30 pairs). As before, this experiment consisted of a training phase followed by a test phase; however, only one type of prey was used, and only one type of vibrational cue, which was generated by dropping of 4.5 ml of sand, was presented. Training consisted of the repeated presentation of the cue followed by prey disappearance. At 2 of the 4 daily feeding times during the training phase, prey was carefully taken from antlions after capture using forceps. Trained antlions were presented the vibrational cue following prey capture but before prey disappearance, whereas untrained antlions were given the cue either 5–10 min before prey presentation or 5–10 min after (randomly selected). Twenty-six antlion pairs were used in the test phase. All of the trained and untrained antlions received prey followed by the cue. We noted whether the captured prey was buried under the sand, and if so, then the time of the beginning and end of the burial within 3 min following cue presentation was recorded.

**Fig. 2:**
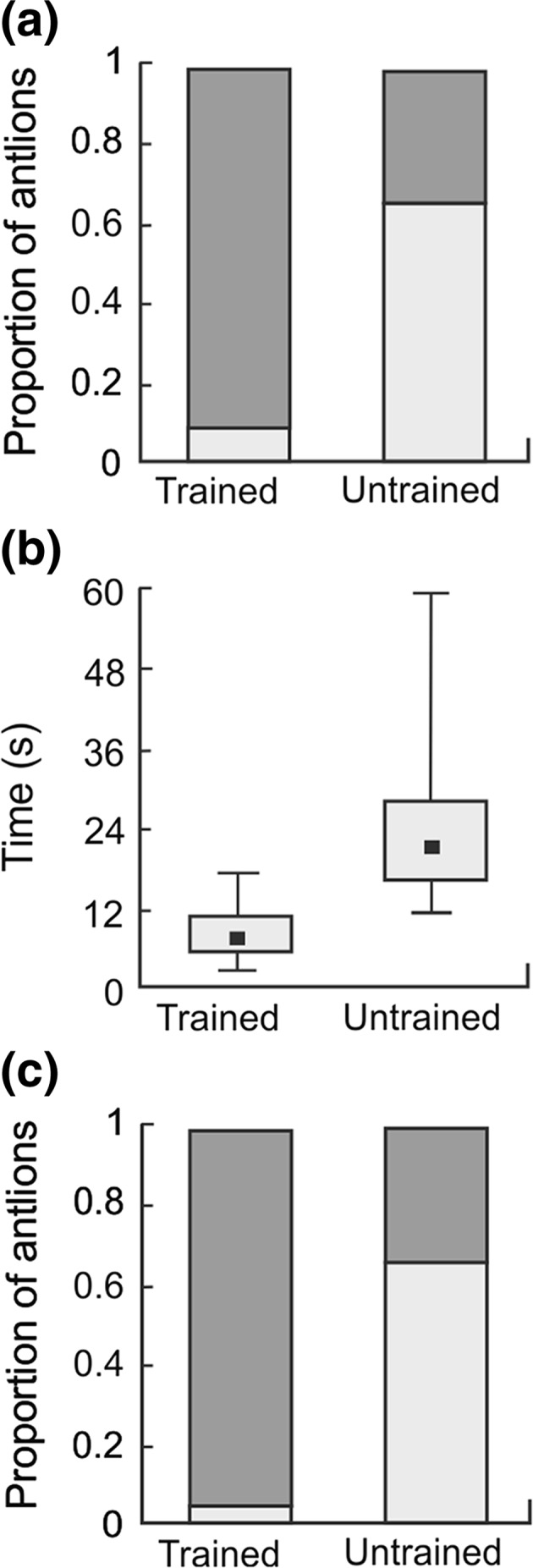
Behaviour of antlions trained to associate a vibrational cue with the loss of prey and untrained antlions with no opportunity to form such an association. **a** Proportion of antlions that initiated burial (*dark grey bars*) and that did not initiate burial (*light grey bars*) during the 3 min following cue presentation; **b** median time (with quartiles and min–max) of the start of prey burial after cue presentation (only individuals who initiated burial are included); **c** proportion of antlions that completely buried (*dark grey bars*) and did not completely bury (*light grey bars*) their prey during the 3 min following cue presentation (only individuals who initiated burial are included)

We found that trained antlions buried their prey more often than did untrained antlions (Fisher’s exact test: *p* = 0.0001). Of the 26 trained antlions, 24 (92 %) initiated prey burial, whereas only 9 (35 %) of the 26 untrained antlions did so (Fig. 2a). Trained antlions also initiated burial sooner (median time of 6 s, quartiles 4–9 s) than did untrained antlions (median at 20 s, quartiles 15–27 s). This difference in the latency to burial was statistically significant (Mann–Whitney *U* test:* U* = 12,* Z* = − 3.87, N1 = 24, N2 = 9, *p* < 0.0001; Fig. 2b). Moreover, prey was more often completely buried by the trained antlions (23 of 24; 96 %) than by the untrained antlions (3 of 9, 33 %; Fisher’s exact test: *p* = 0.0005; Fig. 2c).

## Discussion

The results of Experiment 1 conform to optimal foraging strategy theory, which postulates that fitness will be enhanced by maximizing energy obtained from each prey item while minimizing the energetic costs of hunting (Arnett and Gotelli 2001; Stephens et al. 2007). Previous studies showed that predators usually prefer prey of high profitability. This situation was described for bluegill sunfish (*Lepomis macrochirus*), with individuals preferring large and more profitable prey items over smaller, less profitable prey items under conditions of high prey density (Partridge 1976). Another example comes from great tits (*Parus major*), which can learn the quality of feeding sites and forage in more profitable locations (Werner and Hall 1974). Importantly, the ability to learn positively influences individual fitness: for example, *Biosteres arisanus* wasps, which are egg parasitoids of tephritid fruit flies, were shown to benefit from learning. The learned wasps perceived odour- and colour-related cues associated with potential hosts and parasitized significantly more eggs than did the control, naïve wasps (Dukas 2000). Similar results were shown for newly moulted sixth-instar nymphs of the grasshopper *Schistocerca americana*: nymphs that learned to associate various food qualities with specific cues foraged more efficiently than did unlearned individuals and thus experienced higher growth rates (Dukas and Bernays 2000).

Here, we demonstrated that antlion larvae can greatly benefit from learning cues associated with the presence of large or small prey, as such learning allows them to (1) focus on prey items that are more energetically profitable and (2) fine-tune their foraging strategy to the specific prey type (Experiment 1). Both of these factors may shorten the time needed for development and increase adult body mass, thereby increasing individual fitness due to faster pupation and reproduction (Crowley and Linton 1999; Hollis et al. 2011) as well as reducing larval mortality caused by abiotic (e.g., temperature) and biotic (e.g., predators) factors (Gotelli 1993). Some studies have shown that individuals pupating at lower weights have reduced fitness because their reproductive organs are smaller and because they produce smaller eggs with less fat content (Griffiths 1985). Another advantage of higher larval weight is increased survival during cold winters (northern climates) or rainy seasons (southern climates) compared with the corresponding survival of larvae of lower weights (Griffiths 1985).

Under natural conditions, several circumstances may lead to the loss of caught prey. For example, prey can escape from antlion pit traps, especially in cases involving larger and more energetically profitable prey items (Farji-Brener 2003). Antlions can also lose their prey due to the kleptoparasitic behaviour of other animals (Lucas 1986) and the successful rescue behaviour displayed by ants towards their conspecifics (e.g., Czechowski et al. 2002; Taylor et al. 2013; Miler 2016). Antlion burial under the sand along with its victim has been suggested to be a counter-response to the rescue behaviour of ants (Taylor et al. 2013). Because prey burial is energetically costly to antlions (Fertin and Casas 2006), learning to associate a cue with prey loss can be highly beneficial (Experiment 2). We also do not exclude the possibility that prey burial behaviour can be a form of protection against the formic acid sprayed by ants; the protection conferred by sand has been shown to be decisive in enabling antlions to capture *Camponotus floridanus* ants (Eisner et al. 1993). Similar antlion behaviour was described in the capture of bombardier beetles (Conner and Eisner 1983), which eject hot (100 °C) repellent quinones from the tip of their abdomen when attacked (Aneshansley et al. 1969). Many factors may thus contribute to the prey burial behaviour displayed by antlion larvae, and we do not assume here that one is more important than the others.

In summary, our two experiments demonstrate that antlion larvae are capable of not only learning simple cue–incident associations but also recognizing more complex and interconnected relationships between different stimuli and their relevance, possibly leading to adaptive changes in their behaviour. Our findings provide both new insights into the cognitive abilities of sedentary insects and additional support for the concept of optimal foraging strategies, which emphasizes the importance of maximizing fitness output by balancing the costs and benefits of alternate foraging strategies.

## Electronic supplementary material

Below is the link to the electronic supplementary material.

Supplementary material 1 (DOCX 26 kb)
