# Supplementary material for: Sedentary antlion larvae (Neuroptera: Myrmeleontidae) use vibrational cues to modify their foraging strategies
Source: Anim Cogn. 2016 May 24;19:1037–41. doi: 10.1007/s10071-016-1000-7 (PMC4967082; doi:10.1007/s10071-016-1000-7)
Supplement: Supplementary file 1 — Supplementary material 1 (DOCX 26 kb) [file 10071_2016_1000_MOESM1_ESM.docx]

**Supplementary Methods**

Our study was composed of two parts (Experiments 1 and 2), both of which were conducted at the Institute of Environmental Sciences of Jagiellonian University, Krakow, Poland. Prior to the experiments, larvae of the antlion *Myrmeleon bore* T. were collected from the Bledowska Desert (50°20'46"N; 19°30'48"E) and transported to the laboratory in Krakow, where they were individually housed in paper boxes (13 × 13 × 4.5 cm) that were filled with dry sand (~600 ml in each box). The temperature was maintained at a constant 24°C, and the relative humidity was maintained at between 40 and 60%. Experiment 1 was designed to explore whether antlions can distinguish between the vibrational cues associated with small and large prey and accordingly adapt their foraging strategy to maximize energetic gain. In Experiment 2, we examined whether antlions adopt their behavioural strategy in response to a cue signalling the loss of prey.

*Experiment 1*

Eighty antlion larvae were paired by weight, with one larvae from each pair randomly assigned to the trained treatment or to the untrained control treatment. The experiment consisted of a training phase followed by a test phase: the training phase consisted of 3 blocks, each spanning 3 days (2 consecutive training days followed by a 1-day rest). Trained and untrained antlions were provided with prey in the centre of their pits 4 times per day at 2-hour intervals, with feeding times randomly selected between 10 AM and 6 PM. On each training day, a small prey item was provided at 2 of the 4 feeding times selected at random, and a large prey was provided at each of the other 2 feeding times. Both trained and untrained antlions were fed at the same time. We used *Lasius niger* L. ants as the small prey (head size = 0.829 mm, SD = 0.087, measured as the square root of head width × length) and *Formica polyctena* L. ants as the large prey (head size = 1.719 mm, SD = 0.081, measured as the square root of head width × length). Both the small and large prey were rolled between the experimenter’s fingers for approximately 10 s to stun them before being placed into the pit (Kriete 2014). Prey was delivered immediately following the vibration cue, which lasted for approximately 3 s. Vibration cues were delivered via sand falling into the antlion box from a funnel set on a rack above the box and with a plastic pipette tip (Eppendorf 10 ml) directed towards the edge of the pitfall trap. A small sand collection container (consisting of a metal pipe 4 cm in diameter) below the tip of the pipette prevented additional sand from accumulating in the box and enabled the conduction of vibrations. For trained antlions, we dropped 3 ml of sand as the cue for small prey and 6 ml of sand as the cue for large prey. Untrained (i.e., control) antlions were presented with the same associated cues; however, the cues were presented not directly before prey delivery but either 5 to 10 min before or 5 to 10 min after (randomly selected) the prey were delivered. Therefore, the untrained antlions had no opportunity to learn an association between the cue and the prey size but were habituated to the presence of the cue in their environment. This setup was prepared in accordance with similar setups described in previous research on antlion learning (Guillette et al. 2009; Hollis et al. 2011; Hollis et al. 2015) and is known as the “random control” in Pavlovian conditioning research (sometimes also called the “Explicitly Unpaired Control”; Rescorla 1967).

For the test phase, the 40 trained-untrained pairs of antlions were randomly divided into two groups, each consisting of 18 trained-untrained pairs (4 pairs were excluded due the absence of functional pitfall traps). In the first group, all of the trained and untrained antlions received cues associated with small prey followed by the provision of small ants; after 30 s, during which time the antlions captured their small prey, a second cue associated with large prey was delivered. However, in this first group, no prey were provided to the antlions after the second vibrational cue. In the second group, all of the trained and untrained antlions were treated similarly as in the first group but received large prey preceded by the cue associated with large prey and followed by the cue associated with small prey (after the second cue, no prey was provided). In both groups, we noted whether the captured prey was rejected (i.e., prey thrown away from the pit-trap) or buried under the sand (i.e., downward movement of the larvae along with prey); if either occurred, then we recorded the time of burial or rejection during the 3 min following the delivery of the second cue. For both the prey rejection and burial, we noted the time of behaviour initiation (both can last up to several seconds).

*Experiment 2*

The setup used for the second experiment was similar to that described for Experiment 1. We used naïve 60 weight-matched antlions (not used in the previous experiment), with one member of each pair assigned to the training treatment and the other serving as the untrained control. As with Experiment 1, this experiment consisted of a training phase followed by a test phase; however, only one type of prey was used — *F. polyctena* workers (head size = 1.719 mm, SD = 0.081, measured as the square root of head width × length) — and only one type of vibrational cue was given, which was generated by dropping 4.5 ml of sand. The training phase consisted of 3 blocks, each lasting for 3 days (2 consecutive training days followed by a 1-day rest). Training consisted of the repeated presentation of the cue followed by prey disappearance. At 2 of the 4 daily feeding times during the training phase, the prey was carefully taken from antlions after capture using forceps. Trained antlions were presented with the vibrational cue following prey capture but before prey disappearance, whereas cues presented to the untrained antlions were delivered with no association with prey disappearance, i.e., 5 to 10 min before or 5 to 10 min after (randomly selected) prey capture (‘Explicitly Unpaired Control’; Rescorla 1967). Thus, untrained antlions had no opportunity to form an association between the cue and prey disappearance.

Twenty-six antlion pairs were used in the test phase (4 pairs were excluded due the absence of functional pitfall traps in their boxes). All of the trained and untrained antlions received prey followed by the cue. We noted whether the captured prey was buried under the sand; if so, we recorded the time of the beginning and end of burial during the 3 min following cue delivery. In this experiment, beginning of burial indicates the time at which antlions started to bury themselves and their victims below sand surface (i.e., start of the downward movement of the larvae along with prey), whereas end of burial was indicated by complete prey burial, including ant legs and antennae (i.e., no body parts of ant victim visible above the surface).

*Statistical analysis*

We used Fisher’s exact test to compare the proportions of antlions rejecting and burying their prey between trained and control larvae. It was not possible for antlions to both reject and bury their prey, as these two activities are mutually exclusive. For this reason, we compared the proportion of antlions burying their prey between the two groups, while including only those antlions which did not reject their prey. A Bonferroni correction (P < 0.008) was applied to the analysis for both tests in Experiment 1 because the comparison was among 4 groups (two trained and two untrained). Differences in burying time between trained and untrained antlions in Experiment 2 were tested using the non-parametric Mann–Whitney U test. All calculations were conducted with two-tailed statistical tests and performed using STATISTICA 9.0.

References

Guillette LM, Hollis KL, Markarian A (2009) Learning in a sedentary insect predator: Antlions (Neuroptera: Myrmeleontidae) anticipate a long wait. Behav Processes 80:224–232. doi: 10.1016/j.beproc.2008.12.015

Hollis KL, Cogswell H, Snyder K, et al (2011) Specialized learning in antlions (neuroptera: Myrmeleontidae), pit-digging predators, shortens vulnerable larval stage. PLoS One 6:e17958. doi: 10.1371/journal.pone.0017958

Hollis KL, Harrsch FA, Nowbahari E (2015) Ants vs. antlions: an insect model for studying the role of learned and hard-wired behavior in coevolution. Learn Motiv 50:68–82. doi: 10.1016/j.lmot.2014.11.003

Kriete A (2014) Exploring the link between rescue behavior and experience in the pavement ant, Tetramorium sp. E. PhD Thesis: Mount Holyoke College Sought Hadley, Massachusetts

Rescorla RA (1967) Pavlovian conditioning and its proper control procedures. Psychol Rev 74:71–80.
